# Editorial: The theory and pragmatics of power and relationships in implementation

**DOI:** 10.3389/frhs.2023.1168559

**Published:** 2023-03-23

**Authors:** Erin P. Finley, Svea Closser, Malabika Sarker, Alison B. Hamilton

**Affiliations:** ^1^ VA Greater Los Angeles Healthcare System, Veterans Health Administration, United States Department of Veterans Affairs, Los Angeles, CA, United States; ^2^ Center for Research to Advance Community Health, Joe R. & Teresa Lozano Long School of Medicine, The University of Texas Health Science Center at San Antonio, San Antonio, TX, United States; ^3^ Department of International Health, Bloomberg School of Public Health, Johns Hopkins University, Baltimore, MD, United States; ^4^ Center of Excellence for the Science of Implementation and Scale-Up, BRAC University, Dhaka, Bangladesh; ^5^ Heidelberg Institute of Global Health, University of Heidelberg, Germany; ^6^ Department of Psychiatry and Biobehavioral Sciences, David Geffen School of Medicine, University of California Los Angeles, Los Angeles, CA, United States

**Keywords:** implementation science, theory, implementation practice, power, relationships, implementation strategies, global health

**Editorial on the Research Topic** The theory and pragmatics of power and relationships in implementation

In the early days of implementation science, a series of studies emphasized the essential role of relationships (1, 2), describing features of effective work relationships (3), as well as mechanisms by which those relationships could support effective problem-solving in high-intensity healthcare environments (4–6). In the subsequent decade, however, the field has moved away from social relations to more discrete implementation determinants, strategies, and outcomes (7–9), despite a lingering sense that relationships are an important (if ineffable) factor (10). Along with relationships, power has been largely neglected amid the wealth of implementation theories, models, and frameworks (11), notwithstanding its central importance in change processes and contexts. This Research Topic tackles these gaps in the field, presenting rich examples of implementation efforts from around the world, and specifically examining how they were impacted by local, national, and global relationships and power structures.

Birken et al. start the collection off by identifying organization theories with relevance for implementation science. They identify nine organization theories and more than 70 core constructs, many reflecting gaps in current implementation science theory. They note the particular contribution of organization theory for understanding how power, which they define as “dominance in a relationship,” operates in organizational settings. In doing so they offer tools for understanding how organizational dynamics underlie successes and failures in implementation.

Continuing to explore power, Majumdar et al. draw upon Moon's eight-element taxonomy (12) to explore the strategic deployment of power by diverse agents within the polio eradication initiative in India. Incorporating perspectives from national, regional, local, and frontline agents in the initiative, their analysis demonstrates how power “can be used, shared, or created by actors and networks,” resulting in “opportunities for less powerful actors to wield influence” in often unexpected ways.

Other papers also show that power is neither static nor incontrovertible. In their longitudinal analysis of implementing evidence-informed practices to support LGBTQ + youth in high schools, Shattuck et al. identify how participants were able to “negotiate and incorporate extant power hierarchies.” They point to the critical importance of formative research to understand “the full ecology of the implementation environment,” and to assess where societal and structural pressures may inhibit implementation, particularly in the context of efforts to increase health equity.

Narrowing in on the patient-provider encounter, Mwamba et al. examine clinical interactions occurring across public health facilities in Zambia to explore providers’ uses of discretionary power in implementing patient-centered HIV care. They find that providers worked creatively to reduce hierarchical distance and engage patients as empowered agents in their own care, noting that providers with differing roles and authority (e.g., nurses, medical officers) arrived at differing strategies for engagement based on their positionality and the resources within their control.

Collectively, these papers present an evocative set of case studies, and argue for nuanced and conceptually rich understandings of power relations. Across all of these studies, we find power as both a barrier and a resource, a challenge and a source for improvisation, a halting point and a way of getting things done.

Shifting to the question of relationships, Bartley et al. undertake an analysis of relational theory and the ERIC taxonomy of implementation strategies (9) in order to consider how interactions, exchanges, and alliances operate in each of the most widely recognized implementation strategies. They describe an assessment tool that allows for rating strategies on a continuum from highly relational to highly transactional. In doing so, they illuminate the underexamined role of relationships and power *as an everyday part of implementation*, classifying fully half of the ERIC strategies as highly or semi-relational in nature.

Along parallel lines, Metz et al. directly tackle the question of how relationships can support effective implementation. They present a theoretical model that (1) articulates relational and technical strategies for developing trusting relationships, and (2) hypothesizes pathways linking relationships with implementation outcomes *via* their influence on motivation, capability, and opportunity for change. Their model both furthers the theory of relationships in implementation science and provides guidance for establishing relationships as a foundation for implementation practice.

While developing clinical champions is widely recognized to be a versatile and effective implementation strategy (13, 14), causal pathway mechanisms for how and why champions can be so effective have remained unexplored. Building on existing literature and theory, Morena et al. identify what activities become available to champions in successful implementation relationships. They also note the importance of culture and positionality in identifying potential champions, acknowledging that “without trust and respect, clinical champions yield no influence” and “may experience irreconcilable barriers to implementing change.” Similarly, Hockett Sherlock et al., in their study of nurse-led implementation efforts in three Veterans Health Administration (VHA) hospitals, find that champions’ understanding of both local social networks and power structures is critical to their success. Their article describes how champions “strategically leveraged the structural hierarchy” by encouraging local leaders to show support and facilitate buy-in.

Together, these papers reveal why power and relationships matter in implementation science, and strengthen the theoretical and practical foundations for understanding these dynamics. The contributions of this work come at a critical time for implementation science, as it is poised to move beyond early limitations (15). By directly examining how power and relationships occur in the determinants, strategies, and outcomes of effective implementation, these papers provide a model for implementation research that more incisively balances rigor with engagement and science with pragmatism, increasing the value of the work, and moving us toward a more grounded, relevant, and replicable field (16, 17).

## References

[1] Noël PH Lanham HJ Palmer RF Leykum LK Parchman ML. The importance of relational coordination and reciprocal learning for chronic illness care within primary care teams. Health Care Manage Rev. (2013) 38(1):20–8. 10.1097/HMR.0b013e3182497262 22310483 PMC3383880

[2] Lanham HJ McDaniel RR Crabtree BF Miller WL Stange KC Tallia AF How improving practice relationships among clinicians and nonclinicians can improve quality in primary care. J Comm J Qual Patient Saf. (2009) 35(9):457–66. 10.1016/s1553-7250(09)35064-3 PMC2928073 19769206

[3] Finley EP Pugh JA Lanham HJ Leykum L Cornell JE Veerapaneni P Relationship quality and patient-assessed quality of care in VA primary care clinics: development and validation of the Work Relationships Scale. Annals of Family Medicine. 10.1370/afm.1554 PMC3823725 24218378

[4] Lanham HJ Leykum LK Taylor BS McCannon CJ Lindberg C Lester RT. How complexity science can inform scale-up and spread in health care: understanding the role of self-organization in variation across local contexts. Soc Sci Med. (2013) 93:194–202. 10.1016/j.socscimed.2012.05.040 22819737

[5] Leykum LK Palmer R Lanham H Jordan M McDaniel RR Noël PH Reciprocal learning and chronic care model implementation in primary care: results from a new scale of learning in primary care. BMC Health Serv Res. (2011) 11(1):44. 10.1186/1472-6963-11-44 21345225 PMC3050698

[6] Miller WL McDaniel RR Crabtree BF Stange KC. Practice jazz: understanding variation in family practices using complexity science. J Fam Pract. (2001) 50:872–8. PMID: 11674890

[7] Proctor EK Powell BJ Baumann AA Hamilton AM Santens RL. Writing implementation research grant proposals: ten key ingredients. Implement Sci. (2012) 7(1):96–109. 10.1186/1748-5908-7-96 23062065 PMC3541090

[8] Proctor E Silmere H Raghavan R Hovmand P Aarons G Bunger A Outcomes for implementation research: conceptual distinctions, measurement challenges, and research agenda. Adm Policy Ment Health. (2011) 38(2):65–76. 10.1007/s10488-010-0319-7 20957426 PMC3068522

[9] Powell BJ Waltz TJ Chinman MJ Damschroder LJ Smith JL Matthieu MM A refined compilation of implementation strategies: results from the expert recommendations for implementing change (ERIC) project. Implement Sci. (2015) 10(1):21–35. 10.1186/s13012-015-0209-1 25889199 PMC4328074

[10] Miake-Lye IM Delevan DM Ganz DA Mittman BS Finley EP. Unpacking organizational readiness for change: an updated systematic review and content analysis of assessments. BMC Health Serv Res. (2020) 20(1):106. 10.1186/s12913-020-4926-z 32046708 PMC7014613

[11] Tabak RG Khoong EC Chambers DA Brownson RC. Bridging research and practices: models for dissemination and implementation research. Am J Prev Med. (2012) 43(3):337–50. 10.1016/j.amepre.2012.05.024 22898128 PMC3592983

[12] Moon S. Power in global governance: an expanded typology from global health. Global Health. (2019) 15(S1):74. 10.1186/s12992-019-0515-5 31775779 PMC6881906

[13] Wood K Giannopoulos V Louie E Baillie A Uribe G Lee KS The role of clinical champions in facilitating the use of evidence-based practice in drug and alcohol and mental health settings: a systematic review. Implement Res Pract. (2020) 1:263348952095907. 10.1177/2633489520959072 PMC9924254 37089122

[14] Flanagan ME Plue L Miller KK Schmid AA Myers L Graham G A qualitative study of clinical champions in context: clinical champions across three levels of acute care. SAGE Open Med. (2018) 6:205031211879242. 10.1177/2050312118792426 PMC6075611 30083320

[15] Beidas RS Dorsey S Lewis CC Lyon AR Powell BJ Purtle J Promises and pitfalls in implementation science from the perspective of US-based researchers: learning from a pre-mortem. Implement Sci. (2022) 17(1):55. 10.1186/s13012-022-01226-3 35964095 PMC9375077

[16] Peek CJ Glasgow RE Stange KC Klesges LM Purcell EP Kessler RS. The 5 r’s: an emerging bold standard for conducting relevant research in a changing world. Ann Fam Med. (2014) 12(5):447–55. 10.1370/afm.1688 25354409 PMC4157982

[17] Reed JE Howe C Doyle C Bell D. Simple rules for evidence translation in complex systems: a qualitative study. BMC Med. (2018) 16(1):92. 10.1186/s12916-018-1076-9 29921274 PMC6009041

